# Transfer learning from rating prediction to Top-*k* recommendation

**DOI:** 10.1371/journal.pone.0300240

**Published:** 2024-03-28

**Authors:** Fan Ye, Xiaobo Lu, Hongwei Li, Zhenyu Chen

**Affiliations:** Academy of Computer Science and Technology, Anhui University, Hefei, China; University of Lagos Faculty of Engineering, NIGERIA

## Abstract

Recommender system has made great strides in two major research fields, rating prediction and Top-*k* recommendation. In essence, rating prediction is a regression task, which aims to predict users scores on other items, while Top-*k* is a classification task selecting the items that users have the most potential to interact with. Both characterize users and items, but the optimization of parameters varies widely for their respective tasks. Inspired by the idea of transfer learning, we consider extracting the information learned from rating prediction models for serving for Top-*k* tasks. To this end, we propose a universal transfer model for recommender systems. The transfer model consists of two sub-components: quadruple-based Bayesian Converter (BC) and Prediction-based Multi-Layer Perceptron (PMLP). As the main part, BC is responsible for transforming the feature vectors extracted from the rating prediction model. Meanwhile, PMLP extracts the prediction ratings, constructs the prediction rating matrix, and uses multi-layer perceptron to enhance the final performance. On four benchmark datasets, we use the information extracted from the singular value decomposition plus plus (SVD++) model to demonstrate the effectiveness of BC-PMLP, comparing to classical and state-of-the-art baselines. We also conduct extra experiments to verify the utility of BC, and performance within different parameter values.

## Introduction

Recommender system is an Internet application which is dedicated to studying the users’ interest, item characteristics and other information, and recommending the items that users may be interested in. It is widely used in e-commerce, news media, and content providers [1]. In terms of algorithm research, recommender system mainly solves two problems: rating prediction, which predicts user’s ratings on items that the user have never interacted with, and item sorting, which predicts items’ ranking of the user for recommending top *k* items. There are many studies on rating prediction. From initial content-based collaborative filtering [2, 3], to later collaborative filtering based on matrix factorization(MF) [4], rating predictions are made through historical similarity. The advent of matrix factorization [4] points out a new direction for the recommender system, and many researchers have carried out works on this basis [5, 6]. Subsequently, hybrid recommendation algorithms improved the limitations of evaluation by adding images [7], comments [8–11], geographic [12], etc. [13, 14]. In addition, there are recommendation optimization adopting heterogeneous information networks [15], denoising self-encoder [16, 17], adding emotional analysis [18] and combining matrix factorization [19] with word2vec [20] for rating prediction. With the development of deep learning, matrix factorization based, the deep learning model [21] and the neural network model [22] have obtained excellent performance. In addition, for mixed recommendations, the variety of text analysis [23–25], and the precise analysis of picture information [26] have had a significant impact on subsequent work.

Top-*k*, an issue of item ranking, flourished after the advent of collaborative filtering and matrix factorization [27]. Since S.Rendle et al. [28] proposed a sequence optimization algorithm based on pairwise learning, Bayesian Personalized Ranking(BPR), which can be appropriately applied to the Top-*k* recommendation models of KNN [29] and MF [30], the research on Top-*k* has gradually become diversified. Similarly, Top-*k* recommendation can incorporate other factors into studies [31, 32]. In the direction of heterogeneous information networks, there were also many research methods, such as contextual semantic relevance [33], similarity of heterogeneous information network paths [34], and the attention mechanism [35, 36]. [37] combined the above approaches and suggested a source-path-based context for recommendation using a neural attention model. [38] was a general recommendation model based on heterogeneous information networks to set weights for different entity types. Overall, the above work contributes to the Top-*k* research in various directions.

Essentially, both rating prediction and Top-*k* recommendation model the behavior characteristics of users and items to accomplish their goals. The parameter optimizations vary due to the differences in learning tasks. Our main insight is that the features described by the rating prediction task, should be instructive for Top-*k* recommendation, although they may not be directly applicable to the Top-*k* tasks. In order to explore the guiding significance of rating prediction, we introduce the idea of transfer learning. The simplest way of transferring is sorting the rating results and directly recommending the top *k* items. It is easy to operate, but it has great limitations since the factors affecting the users’ selections are extremely complex, not only the ratings. In the consideration of the strong correlation between the two tasks, we try to extract the information learned from the advanced work of rating prediction task, and apply it for Top-*k* recommendation. To this end, we propose a BC-PMLP model, which is capable to transform rating prediction into Top-*k* classification, that is, the information learned in the rating prediction task is transferred and converted, so that the label space is converted from the rating to the interaction possibility. The model consists of two parts: quadruple-based Bayesian Converter (BC) and Prediction-based Multi-Layer Perceptron (PMLP). Firstly, we extract feature vectors and prediction ratings from a rating prediction model (called the underlying model) as the input of BC-PMLP. The main part BC draws on the idea of BPR-MF, and adopts a more advanced quadruple training method for training, which will transform original vectors for learning implicit interaction information. At the same time, PMLP extracts the prediction ratings, uses the square loss between the explicit rating and the output value for optimization, and adopts a multi-layer perceptron for concentrating on explicit ratings. The two parts will be combined through a balance factor, which can well reflect the proportion between implicit interactions and explicit ratings to achieve better transfer learning performance.

Overall, the contributions of this paper can be summarized as follows:

- We propose to use the transfer learning idea to link the rating prediction and Top-*k* tasks, and give a specific definition of the inductive transfer learning.
- We propose the BC-PMLP consisting of Bayesian Converter, Prediction based Multi-Layer Perceptron and a balance factor, to realize transfer learning. It also contains more novel methods such as quadruple training and dynamic sampling, which can be independently applied to other algorithms.
- We verified the effectiveness of BC-PMLP and proposed methods with many experiments. Only from the experimental results, our work makes the Top-*k* recommendation task more satisfactory, which means that the application of transfer learning idea is successful.

The rest of the paper is organized as follows. Section Related work presents related work for reference. Section Preliminaries gives the preliminary definition of our work. Section Methods describes the proposed BC-PMLP in detail, including Bayesian Converter, Prediction-based Multi-Layer Perceptron and some other fusion details. BC is responsible for transforming the feature vectors extracted from the rating prediction model. PMLP extracts the prediction ratings, constructs the prediction rating matrix, and uses multi-layer perceptron to enhance the final performance. BC and PMLP are then better fused by a balance factor. And some experimental results are shown and discussed in Section Experiments. And finally give the conclusion of this paper in Section Conclusion.

## Related work

This section will first introduce some related work on translating rating predictions into Top-*k* recommendations. In recent years, the research of recommender systems has developed rapidly, especially in the regression task [7, 21, 39] of rating prediction, the effect is especially outstanding. One of the most classic is singular value decomposition (SVD), which was later developed to SVD++ [40](this will also be the basis for our follow-up work.). SVD++ adds the user bias information and implicit parameters to describe user preferences, and calculate ratings by the following equation:

$$\begin{array}{c} {\hat{r}}_{ui}=\mu +b_{i}+b_{u}+{\text{q}}_{i}^{T}({\text{p}}_{u}+|I_{u}{|}^{-\frac{1}{2}}\sum_{j\in I_{u}^{+}}{\text{y}}_{j}) \end{array}$$

where *μ*, *b*_*i*_ and *b*_*u*_ represent the mean of global ratings, the user bias and the item bias respectively, and **y** is the implicit intersection feedback of $I_{u}^{+}$. With this equation, SVD++ has achieved better effect in rating prediction.

Relatively, the performance of Top-*k* tasks is slightly inferior to rating prediction. Implicit feedback based collaborative filtering and matrix factorization are the two cornerstones of Top-*k* task, on which fruitful work such as NCF [22], NGCF [41] and LightGCN [42] have grown. NCF improved the recommended algorithm using the multi-layer sensor fusion generalized matrix decomposition. NGCF adopts GNN layers on the user-item interaction graph, which exploits the user-item graph structure by propagating embeddings on it to refine user and item representations. LightGCN removes the feature transformation and non-linear activation in NGCF and improved both performance and efficiency. Also, researchers always try to collect more information to describe users and items more completely. For example, in SVAE [43], time-series information is used to predict the most likely interactive items in the next period of time based on the user’s interaction in a known time period. Hsieh et al. studied the relationship between metric learning and collaborative filtering and proposed collaborative metrical learning (CML) [44] to learn the joint metrics space, which reveals the bottom range of user fine-grained preferences well. In addition, variational autoencoders (VAEs) have gained attention as depth generation models with their ability to approximate data distribution. RecVAE [45] is based on the variational autoencoder that reconstructs partially-observed user vectors, which introduces several techniques to improve M-VAE. JoVA-H [46] is an ensemble of two VAEs to jointly learn both user and item representations to predict user preferences.

In real life, Top-*k* application scenarios [27, 31, 32] are more extensive. Our key insight is the guiding significance of advanced rating prediction for Top-*k* recommendation. We introduce the idea of transfer learning to link the existing rating prediction regression with Top-*k* sorting. The easiest consideration is to put the user sequence directly into the prediction model, get each user’s prediction score of each item, and select *k* items with the highest rating among all items. The poor performance validates that users’ interactions are always influenced by complex factors, not just ratings. Therefore, we should utilize and process the features learned from rating model(often appear as use and item embeddings) to serve the Top-*k* task, instead of directly using the rating predictions for recommendation. Other auxiliary parameters are trained specifically for rating prediction tasks, so they are not suitable to be transferred. As a result, we must adopt algorithms that work well with feature vectors without introducing additional parameters. BPR-MF [28] catches our eye because of its milepost contribution of representing users and items. BPR selects one non-observed item *j* as the negative observation for an observed interaction (*u*, *i*), and generates a learning pair (*u*, *i*, *j*). (*u*, *i*, *j*) contains a total order *i* >_*u*_ *j*, which means *u* prefers *i* to *j*. By strengthening the total order >_*u*_, *u* is more inclined to *i* than before. Since the final ranking performance only depends on feature vectors, we believe BPR has reliable ability for processing feature vectors.

Although the predicted ratings cannot be used directly for ranking, they also make beneficial effect on Top-*k* task. An excellent model in Top-*k* recommendation, DMF [21], refers to the rating information as explicit ratings, and references both implicit feedback and explicit ratings during the input phase. Inspired by the deep structured semantic models, DMF constructed a neural network structure to learn a common potential low-dimensional space to represent users and items. Its advancement inspires us with the potential of transferring ratings. Table 1 provides more details to analyze the attributes and contributions of these works.

**Table 1:** Overview of related work.

Task	Work	Contribution
rating prediction	SVD	The user’s score data is a sparse matrix, which can be mapped to low dimensional space by SVD.
	SVD++	SVD++ adds the user bias information and implicit parameters to describe user preferences.
Top-*k*	NCF	NCF improves the recommended algorithm using the multi-layer sensor fusion generalized matrix decomposition.
	NGCF	NGCF adopts GNN layers on the user-item interaction graph, which exploits the useritem graph structure by propagating embeddings on it to refine user and item representations.
	LightGCN	LightGCN removes the feature transformation and non-linear activation in NGCF and improved both performance and efficiency.
	SVAE	In SVAE, time-series information is used to predict the most likely interactive items in the next period of time based on the user’s interaction in a known time period.
	CML	CML learns a metric space to encode the user-item interactions and to implicitly capture the user-user and item-item similarities.
	BPR-MF	BPR is a matrix factorisation method that optimises a pairwise ranking function using negative sampling, through stochastic gradient descent.
	DMF	DMF refers to the rating information as explicit ratings, and references both implicit feedback and explicit ratings during the input phase.
	RecVAE	RecVAE is based on the variational autoencoder that reconstructs partially-observed user vectors, which introduces several techniques to improve M-VAE.
	JoVA-H	JoVA-H is an ensemble of two VAEs to jointly learn both user and item representations to predict user preferences.
	BC-PMLP(ours)	We propose the BC-PMLP consisting of Bayesian Converter, Prediction based Multi-Layer Perceptron and a balance factor, to realize transfer learning.

Overall, transfer learning for recommender system faces two main challenges:

- The first challenge is the definition of transfer learning in recommender system. This issue mainly includes what information should be transferred and how the information is processed. The solutions determine the transfer algorithm.
- The second challenge is to find out whether both explicit ratings and implicit interactions play an important role in the transfer process, and how to perform different transfer treatments and combine the two.

To this end, we give the theoretical basis of our proposed transfer algorithm in the following and propose BC-PMLP for transfer learning from rating prediction to Top-*k* recommendation. We make the BC and PMLP receive implicit feedback and explicit rating information respectively. The BPR-MF pairwise learning method is improved and optimized in the BC, named quadruple training method, to learn implicit interactions. PMLP receives explicit rating information and uses multi-layer perceptron for training. Finally, these two parts will be combined by a balance factor to achieve better transfer learning performance. Before introducing in detail, we will define transfer learning in recommender system.

### Preliminaries

In this section, we will focus on the preliminary definition of transfer learning from rating prediction problem to Top-*k* task. The question of rating prediction is how you predict unknown user ratings from known user history. In Top-*k* recommendation, K items are recommended to the user, and these recommendations are presented to the user in descending order based on the user’s “rating” of the item. For example, when you browse Amazon, the site will recommend K items that you are most likely to buy. Transfer learning is a machine learning method that transfers the knowledge learned through $T_{s}$ tasks in the source domain to $T_{t}$ tasks in the target domain to improve the performance of $T_{t}$ task model prediction. The task of transfer learning is to start from the similarity, find the similarity of the target problem, and apply the model learned in the old domain to the new domain. Transfer learning is common for humans, for example, we might find that learning to recognize cars might help identify trucks, or learning to play the electronic organ might help learn the piano. Transfer learning involves concepts of source domain and target domain, which are rarely mentioned in recommender system. So, we first give the definitions of the two domains in this paper:

**Definition 1**. **(Source Domain: rating prediction)** Given the feature space X, and the data distribution *P*_*s*_(*X*), both constitute source domain, denoted as: $D_{s}≔\left\{X,P_{s}\left(X\right)\right\}$. The corresponding learning task is denoted as: $T_{s}≔\left\{R,f_{r}\left(\cdot \right)\right\}$, where R and *f*_*r*_(⋅) are label space and rating prediction function respectively.

**Definition 2**. **(Target Domain: Top-*k* recommendation)** Given the feature space X, and the data distribution *P*_*t*_(*X*), both constitute target domain, denoted as: $D_{t}≔\left\{X,P_{t}\left(X\right)\right\}$. The corresponding learning task is denoted as: $T_{t}≔\left\{Y,f_{t}\left(\cdot \right)\right\}$, where Y and *f*_*t*_(⋅) are label space and classification function respectively.

On this basis, we will give the specific definition of transfer learning in our work below.

**Definition 3**. **(Transfer Learning for Recommender System)** Given a source domain $D_{s}$ and learning task $T_{s}$, a target domain $D_{t}$ and learning task $T_{t}$, where $D_{s}$ and $D_{t}$ have the same feature space. Transfer learning aims to modify the data distribution from *P*_*s*_(*X*) to *P*_*t*_(*X*), and learn a new predictive function *f*_*t*_(⋅) to generate the binary classification task labels of 0 or 1 for the Top-*k* task.

According to these definitions, we can get Top-*k* results based on an implemented rating prediction model.

## Methods

In this section, we will give introduction for the proposed transfer model for recommender system. We first introduce the data sources and then the Bayesian Converter, which converts feature vectors with a quadruple training method. Next, a Multi-Layer Perceptron based on prediction is discussed in detail. Finally, we present the overall structure of our proposed transfer model, BC-PMLP, including some combination details.

### Dataset descriptions

In our experiments, we selected four real-world datasets which have been widely used in other recommender systems: MovieLens-1m (ML-1m), Netflix, FilmTrust and Yelp. We use such four data sets to evaluate the effectiveness of our methods.

#### 1.Movielens-1M

The Movielens-1M dataset contains user rating and review data for movies, as well as basic information about users and movies. Movielens-1M is a public dataset available at https://grouplens.org/datasets/movielens/1m/. It contains 1000209 records from 6040 users for 3706 movies, which is a record of interactions between users and movies. Select data on the interaction record between users and movies (including userID, itemID, ratings, and timestamps). We use the Movielens-1M original dataset for experiments. The main part used for our experiments is the file ratings.dat.

#### 2.Netflix

Netflix is the user-movie rating data from the Netflix Prize. This is a public dataset available at https://www.kaggle.com/datasets/netflix-inc/netflix-prize-data. The data were collected between October, 1998 and December, 2005 and reflect the distribution of all ratings received during this period. The ratings are on a scale from 1 to 5 (integral) stars. Select data on the interaction record between users and items (including userID, itemID, ratings, and timestamps). For Netflix, we created a sample which consists all interactions related to 5000 items. The main parts used for our experiments are the two files combined_data_1.txt and combined_data_2.txt.

#### 3.FilmTrust

FilmTrust is a small dataset crawled from the entire FilmTrust website in June, 2011. It is public available at https://guoguibing.github.io/librec/datasets.html. It contains 35497 data records from 1508 users for 2071 items, which is a dataset for recording interactions between users and movies through ratings. Select data on the interaction record between users and items (including userID, itemID, and ratings). We use the FilmTrust original dataset for experiments. The main part used for our experiments is the file ratings.txt.

#### 4.Yelp

The Yelp dataset contains data such as users’ personal information, basic information about businesses, and users’ comments and ratings on businesses. Yelp is public available at https://github.com/hexiangnan/sigir16-eals/tree/master/data. Wherein, the local businesses like restaurants and bars are viewed as the items. Select data on the interaction record between users and items (including userID, itemID, ratings, and timestamps). As preprocessing for Yelp, we filtered out the users who had less than 60 ratings and the items that were rated by less than 60 users. The main part used is the file yelp.rating.

The datasets were originally collected in line with the terms and conditions of the data holder.Some statistics are shown in Table 2. Note that the original dataset only contains the following information: ids of user and item, ratings and timestamps. All baselines can only use this information.

**Table 2:** The detailed information of four datasets.

Dataset	users	items	interactions	density
MovieLens-1m	6040	3706	1000209	4.47%
Netflix	9627	5000	1476391	3.07%
FilmTrust	1508	2071	35497	1.14%
Yelp	742	2741	77589	3.81%

For each dataset, we randomly select 80% of historical interactions of each user to constitute the training set, and treat the remaining as the test set. The minimal data set necessary to replicate our finding can be found in https://www.kaggle.com/datasets/lvancn/plos-data.

### Bayesian Converter (BC)

As discussed above, the core part of the whole transfer learning process is transformation of the feature distribution, by which original feature vectors serving rating prediction tasks can be transformed into the vectors suitable for Top-*k* tasks. Therefore, we consider that the pairwise learning method cited in [28] which strengthening the total order >_*u*_ of (*u*, *i*, *j*), has a significant ability to transform the vectors.

According to the principle of BPR, we analyze the influence of *i* and *j* on updating *u* and get the conclusion that they are equal. It’s not reliable, because the selection of negative item *j* is random, and the influence of *j* on *u* is difficult to judge accurately in complex situation. To this end, we analyze training pairs from another point of view, considering that *i* chooses *u* instead of u chooses i, so positions of *u* and *i* are equal in training pairs. Corresponding to the negative item, we add an extra negative user, *v*, which has non-observed to *i*, into the learning pair to compose a quadruple. According to the same status of *u* and *i*, *j* and *v* in the interaction relationship, the derivation process of quadruple training method will be given as follows.

Firstly, as mentioned above, we define the quadruple and the training dataset as follows:

$$\begin{array}{c} quadruple≔(u,i,j,v) \end{array}$$ (1)

$$\begin{array}{c} T_{BC}≔\{\left(u,i,j,v\right)|i\in I_{u}^{+}\wedge j\in I\backslash I_{u}^{+}\wedge v\in U\backslash U_{i}^{+}\} \end{array}$$ (2)

where *I* and *U* represent the set of items and users respectively. $I_{u}^{+}$ is the collection of items observed by a user *u*, and $U_{i}^{+}$ is the collection of users observed by a item *i*. Then, the total order relation *i* >_*u*_ *j* is defined to indicate that *u* prefers *i* than *j*, and *u* >_*i*_ *v* indicates that *u* is more likely to choose *i* than *v*. Both of them meet the properties of totality, antisymmetry and transitivity. Assume that all users, items, interactions, and generated learning pairs are independent of each other, according to Bayesian formulation, the following derivations are obtained:

$$\begin{array}{c} P\left(\theta_{1}|{>}_{u}\right)=\frac{P\left({>}_{u}|\theta_{1}\right)*P\left(\theta_{1}\right)}{P({>}_{u})},P\left(\theta_{2}|{>}_{i}\right)=\frac{P\left({>}_{i}|\theta_{2}\right)*P\left(\theta_{2}\right)}{P({>}_{i})} \end{array}$$ (3)

$$\begin{array}{c} P\left(\theta_{1}|{>}_{u}\right)\propto P\left({>}_{u}|\theta_{1}\right)*P\left(\theta_{1}\right),P\left(\theta_{2}|{>}_{i}\right)\propto P\left({>}_{i}|\theta_{2}\right)*P\left(\theta_{2}\right) \end{array}$$ (4)

where *θ*_1_, *θ*_2_ represents the parameter vectors, and the posterior probability *P*(*θ*_1_| >_*u*_), *P*(*θ*_2_| >_*i*_) are the maximizing target. According to totality and antisymmetry of total orders, the user-specific likelihood function can be simplified to:

$$\begin{array}{c} \prod_{u\in U}P({>}_{u}|\theta_{1})=\prod_{\left(u,i,j,v\right)\in T_{s}}P(i{>}_{u}\;j|\theta_{1}) \end{array}$$ (5)

$$\begin{array}{c} \prod_{i\in I}P({>}_{i}|\theta_{2})=\prod_{\left(u,i,j,v\right)\in T_{s}}P(u{>}_{i}\;v|\theta_{2}) \end{array}$$ (6)

As we have explained before, quadruple training method is an enhanced version of pairwise method. Therefore, we adopt the dot product of the two and the logistic sigmoid function to define the individual probability:

$$\begin{array}{c} P\left(i{>}_{u}j|\theta_{1}\right)≔\sigma \left({\hat{x}}_{uij}\left(\theta_{1}\right)\right),P\left(u{>}_{i}\;v|\theta_{2}\right)≔\sigma \left({\hat{x}}_{iuv}\left(\theta_{2}\right)\right) \end{array}$$ (7)

$$\begin{array}{c} {\hat{x}}_{uij}≔{\hat{x}}_{ui}-{\hat{x}}_{uj},{\hat{x}}_{iuv}≔{\hat{x}}_{iu}-{\hat{x}}_{iv} \end{array}$$ (8)

$$\begin{array}{c} {\hat{x}}_{ui}≔{\text{p}}_{u}⊙{\text{q}}_{i},{\hat{x}}_{uj}≔{\text{p}}_{u}⊙{\text{q}}_{j},{\hat{x}}_{iv}≔{\text{q}}_{i}⊙{\text{p}}_{v} \end{array}$$ (9)

where the **p**_*u*_, **q**_*i*_, **q**_*j*_, **p**_*v*_ are the representation vectors of *u*, *i*, *j*, *v* whose initial distribution obeys *P*_*s*_. In the following, we can formulate the maximum logarithmic posterior estimator to derive generic optimization criterion:

$$\begin{array}{c} \begin{array}{cc} OPT & ≔lnP(\theta |>)\\ & =lnP(\theta |>)P(\theta )\\ & =ln\prod_{\left(u,i,j,v\right)\in T_{s}}\sigma \left({\hat{x}}_{ui}-{\hat{x}}_{uj}\right)*\sigma \left({\hat{x}}_{iu}-{\hat{x}}_{iv}\right)+lnP\left(\theta \right)\\ & =\sum_{\left(u,i,j,v\right)\in T_{s}}\left(ln\sigma \left({\hat{x}}_{uij}\right)+ln\sigma \left({\hat{x}}_{iuv}\right)\right)-\lambda_{\theta }{\left||\theta |\right|}^{2} \end{array} \end{array}$$ (10)

Note that *θ* and > are the general term of *θ*_1_ and *θ*_2_, >_*u*_ and >_*i*_. λ_*θ*_ is the regularization parameters. According to the criterion of stochastic gradient descent, the updating process is given as follows:

$$\begin{array}{c} \begin{array}{cc} \frac{\partial OPT}{\partial \theta }= & \sum_{\left(u,i,j,v\in T_{s}\right)}\left(\frac{\partial }{\partial \theta }ln\sigma \left({\hat{x}}_{uij}\right)+\frac{\partial }{\partial \theta }ln\sigma \left({\hat{x}}_{iuv}\right)\right)-\lambda_{\theta }\frac{\partial }{\partial \theta }{\left||\theta |\right|}^{2}\\ \propto & \sum_{\left(u,i,j,v\in T_{s}\right)}\frac{-e^{-{\hat{x}}_{uij}}}{1+e^{-{\hat{x}}_{uij}}}\cdot \frac{\partial }{\partial \theta }{\hat{x}}_{uij}-\frac{e^{-{\hat{x}}_{iuv}}}{1+e^{-{\hat{x}}_{iuv}}}\cdot \frac{\partial }{\partial \theta }{\hat{x}}_{iuv}-\lambda_{\theta }\theta \end{array} \end{array}$$ (11)

and proceed to the final step:

$$\begin{array}{c} \theta \leftarrow \theta +\alpha (\frac{1}{1+e^{{\hat{x}}_{uij}}}\cdot \frac{\partial }{\partial \theta }{\hat{x}}_{uij}-\frac{1}{1+e^{{\hat{x}}_{iuv}}}\cdot \frac{\partial }{\partial \theta }{\hat{x}}_{iuv}-\lambda_{\theta }\theta ) \end{array}$$ (12)

$$\begin{array}{c} \left\{ \begin{array}{cc} \frac{\partial OPT}{\partial u} & =\frac{1}{1+e^{{\hat{x}}_{uij}}}\cdot \left({\text{q}}_{i}-{\text{q}}_{j}\right)+\frac{1}{1+e^{{\hat{x}}_{iuv}}}\cdot {\text{q}}_{i}-\lambda {\text{p}}_{u}\\ \frac{\partial OPT}{\partial i} & =\frac{1}{1+e^{{\hat{x}}_{uij}}}\cdot {\text{p}}_{u}+\frac{1}{1+e^{{\hat{x}}_{iuv}}}\cdot \left({\text{p}}_{u}-{\text{p}}_{v}\right)-\lambda {\text{q}}_{i}\\ \frac{\partial OPT}{\partial j} & =-\frac{{\text{p}}_{u}}{1+e^{{\hat{x}}_{uij}}}-\lambda {\text{q}}_{j}\\ \frac{\partial OPT}{\partial v} & =-\frac{{\text{q}}_{i}}{1+e^{{\hat{x}}_{iuv}}}-\lambda {\text{p}}_{v} \end{array}\right. \end{array}$$ (13)

For facilitating comparison, we restore learning pair to triples, (*u*, *i*, *j*), and derive the updating process of pairwise parameters as follows:

$$\begin{array}{c} \left\{ \begin{array}{cc} \frac{\partial BPR}{\partial u} & =\frac{1}{1+e^{{\hat{x}}_{uij}}}\cdot \left({\text{q}}_{i}-{\text{q}}_{j}\right)-\lambda {\text{p}}_{u}\\ \frac{\partial BPR}{\partial i} & =\frac{{\text{p}}_{u}}{1+e^{{\hat{x}}_{uij}}}-\lambda {\text{q}}_{i}\\ \frac{\partial BPR}{\partial j} & =-\frac{{\text{p}}_{u}}{1+e^{{\hat{x}}_{uij}}}-\lambda {\text{q}}_{j} \end{array}\right. \end{array}$$ (14)

The meaning of variables in the formulation is the same as before. By contrast, we can observe that to update *u*, coefficient ratio of **q**_*i*_ and **q**_*j*_ in the quadruple is larger than which in pairwise method, which means we expand the influence of positive item *i* on *u*. In addition, during the process of parameter updating, the feature vector of user *v* is involved synchronously, which improves the final performance and convergence speed of parameter updating.

As the main part of BC, quadruple training method will convert feature vectors extracted from the underlying model. In other words, feature vectors are extracted as the initialization of user and item vectors in BC, and the quadruple training method is adopted for vector transformation. Finally, the calculation of interaction probability between *u* and *i* is given as follows:

$$\begin{array}{c} {\hat{y}}_{ui}=F^{BC}\left(u,i|{\text{p}}_{u},{\text{q}}_{i}\right)=\sigma \left({\text{p}}_{u}⊙{\text{q}}_{i}\right) \end{array}$$ (15)

As one of the most prominent contributions of this paper, BC is capable to take the information provided by the rating prediction model and convert it into embeddings suitable for Top-*k*. Meanwhile, the quadruple training method can also be independently used to replace the pairwise learning method in other algorithms, and subsequent experiments will verify this contribution.

If we follow the design of BPR-MF, we can already get the ranking by taking the dot product of vectors. But we still propose the Multi-Layer Perceptron based on prediction in the following for better use of transferred ratings.

### Prediction based Multi-Layer Perceptron (PMLP)

Generally, most of Top-*k* models are based on the implicit feedback matrix, in which values are binarized 1 or 0 denoting whether *u* has interacted with *i* or not. It is mentioned in [21] that explicit ratings, continuous predicted values in the interval from 0 to 5, can be combined with implicit feedback in one model by a new designed loss function. The construction of DMF inspired us that transferable explicit ratings may have great potential to enhance the final performance, although we have already achieved the goal of transfer learning with BC.

For this purpose, referring to the work in [22], we construct a Multi-Layer Perceptron (MLP) to transfer the ratings. Differently, we directly adopt explicit ratings for matrix construction instead of binarized ratings, and the corresponding loss function is also changed from the cross entropy to the square loss. Our novel design is setting non-observed interaction values with prediction ratings, outputs of the underlying model, instead of 0 in [21], and we name this design Prediction based Multi-Layer Perceptron(PMLP).

Specifically, the interaction between *u* and *i* in prediction matrix is defined as:

$$\begin{array}{c} r_{ui}=\left\{ \begin{array}{cc} & {\text{R}}_{ui}^{GT},\;if\;interaction\;is\;observed;\\ & {\text{R}}_{ui}^{PR},\;otherwise. \end{array}\right. \end{array}$$ (16)

where ${\text{R}}^{GT}\in R^{M\times N}$ and ${\text{R}}^{PR}\in R^{M\times N}$ denote ground truth matrix and predicted rating matrix(transferred from the underlying model) respectively. Each element is contained within the label space R. Subscript *ui* represents the element of row *u* and column *i* in the matrix, i.e. the rating scoring by *u* for *i*. Note that **R**^*GT*^ is a sparse matrix composed of observed interactions, and elements in **R**^*PR*^ are outputs of the underlying model.

**Fig 1:**
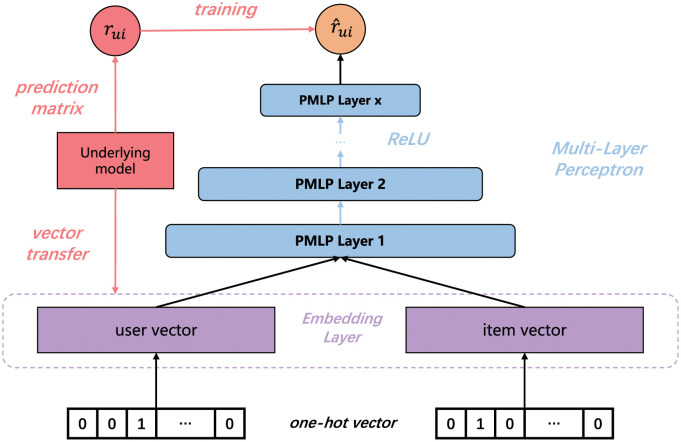
The structure of PMLP. *u* and *i* represent user and item. Subscript *ui* represents the element of row *u* and column *i* in the matrix. *r*_*ui*_ represents the interaction between *u* and *i* in prediction matrix. ${\hat{r}}_{ui}$ is the final output.

For a more intuitive understanding, Fig 1 shows the overall structure of proposed PMLP. The bottom input layer consists of one-hot vectors representing users and items, which will be used to project sparse representation into dense vector in the embedding layer. Particularly worth mentioning we initialize the embedding layer with transferred vectors from the underlying model like the design in BC, instead of random initialization. The embedding participates synchronously in updating process of PMLP, which indicates that PMLP also has the ability of modifying feature distribution. Immediately after that, we concatenate the output of embedding layer, **p**_*u*_ and **q**_*i*_, as the input of the fully connected layer. Precisely, the Prediction based Multi-Layer Perceptron (PMLP) is defined as:

$$\begin{array}{c} \begin{array}{c} v=a\left({\text{p}}_{u},{\text{q}}_{i}\right)=[\begin{array}{c} {\text{p}}_{u}\\ {\text{q}}_{i} \end{array}],\\ v_{l}=a_{l}(\ldots a_{2}({\text{W}}_{2}^{T}a_{1}\left({\text{W}}_{1}^{T}v+{\text{b}}_{1}\right)+{\text{b}}_{2})\ldots ),\\ {\hat{r}}_{ui}={\text{h}}^{T}{\text{v}}_{l} \end{array} \end{array}$$ (17)

where *a*_*x*_, **W**_*x*_ and **b**_*x*_ denote the activation function, weight matrix and bias vector for the *x*-th layer’s perceptron, respectively. Function *a* represents the concatenation of **p**_*u*_ and **q**_*i*_, and *l* is the number of layers. According to previous work experience, as shown in Fig 1, the network structure is designed as a tower pattern, where the bottom layer is the widest, and we halve the layer size for each successive higher layer. Meanwhile, Rectifier (ReLU) is adopted as the activation function empirically. In this formulation, **v**_*l*_ is the output of the last fully connected layer, and **h** is the weight vector of the prediction layer. The final output, ${\hat{r}}_{ui}$, will take *r*_*ui*_ as the target value and square loss as the loss function to update parameters of entire model.

### Fusion of BC and PMLP

As mentioned above, BC pays attention to implicit interactions, while PMLP pays attention to explicit ratings. Both modify the feature distribution from *P*_*s*_ to *P*_*t*_, and output two values, ${\hat{y}}_{ui}$ and ${\hat{r}}_{ui}$, which can measure the interaction from different perspectives. In order to reduce the complexity of the entire model while combining the two values, we set a balance factor, *α*, which balances the weight of ${\hat{y}}_{ui}$ and ${\hat{r}}_{ui}$. The final interaction calculation is given as follows:

$$\begin{array}{c} z_{ui}=\alpha *{\hat{y}}_{ui}+\left(1-\alpha \right)\frac{{\hat{r}}_{ui}}{max(R)} \end{array}$$ (18)

where *max*(*R*) denotes the upper limits of ratings (5 in a 5-star system), which is adopted for normalization. Finally, the prediction function *f*_*t*_(⋅) can be defined as:

$$\begin{array}{c} f_{t}\left(u,i\right)=\left\{ \begin{array}{cc} & 1,\;if\;z_{ui}\;is\;one\;of\;the\;highest\;top\;k\;ratings;\\ & 0,\;otherwise. \end{array}\right. \end{array}$$ (19)

**Fig 2:**
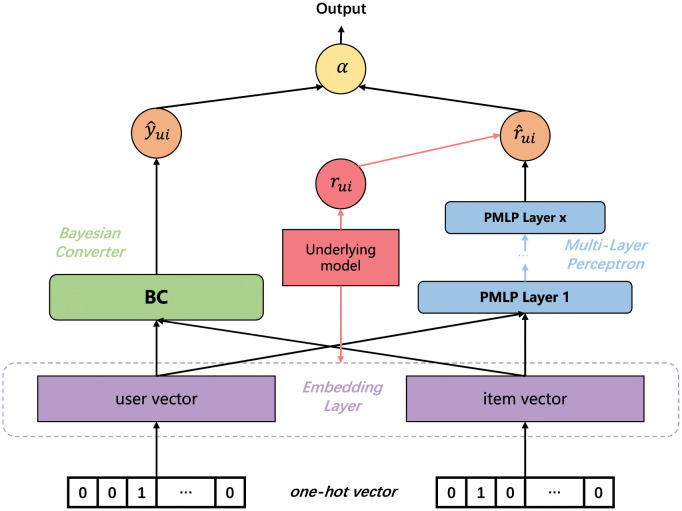
The overall structure of BC-PMLP. *u* and *i* represent user and item. Subscript *ui* represents the element of row *u* and column *i* in the matrix. *r*_*ui*_ represents the interaction between *u* and *i* in prediction matrix. ${\hat{r}}_{ui}$ is the final output. ${\hat{y}}_{ui}$ represents the interaction probability between *u* and *i* after the Bayesian Converter layer processing.

The structure of the entire model is shown in Fig 2, and each of specific steps has been explained in detail in the previous section. In addition, there are several points that need to be specified:

- The training process for BC and PMLP involves the formation of training pairs, that is, the selection of negative samples. In other work, the number of negative samples is fixed, for example, it is set to 4 as [22] for each user-item interaction. However, in recommender systems, excessive training causes not only over-fitting, but also mistaking positive samples as negative samples. In this paper, we design a dynamic sampling method, which determines the number of negative samples based on the observed user interaction records. Specifically, for each user, the numbers of positive samples on training set and test set, are denoted as *m*_*tr*_ and *m*_*te*_ respectively, and the total number of items is denoted as *N*. Suppose *n* sampling is performed for each interaction, and the number of mistaken positive sample is denoted as variable *X*. Then *X* follows the hypergeometric distribution with parameters *n*, *m*_*te*_ and *N*, denoted as *X* ∼ *H*(*n*, *m*_*te*_, *N*). Since interactions on the test set is not visible during training, in order to prevent the *m*_*te*_ positive samples from being sampled at random, we set the expectation of *X* is less than 1. According to the expectation formula, the following results can be obtained:
  $$\begin{array}{c} E\left(X\right)=n*m_{tr}\frac{m_{te}}{N}\leq 1\;\Leftrightarrow \;n\leq \frac{N}{m_{te}*m_{tr}} \end{array}$$ (20)
  Since *m*_*te*_ is unknown during sampling, we use *per* * *m*_*tr*_ to estimate *m*_*te*_, where *per* is the percentage of the test set in the full dataset. Therefore, the sampling number for each interaction is:
  $$\begin{array}{c} n=\frac{N}{per*m_{tr}^{2}} \end{array}$$ (21)
  Based on this dynamic sampling method, the model can take both training efficiency and effect into account.
- As mentioned before, in SVD++, a factor vector is associated with item *i*, denoted as **y**_*i*_, which is a supplement to the user’s factor preference from the perspective of implicit feedback, and the representation vector is denoted as **q**_*i*_. In the process of extracting vectors, we concatenate **y**_*i*_ and **q**_*i*_, which resulted in the dimension of item vectors being exactly twice that of user vectors. To this end, we perform an additional process on the item vector to halve its dimension, to achieve the goal of equal dimensions of user and item vectors. Simply, we add the even-numbered dimension of the concatenated vector to the odd-numbered dimension, and then delete the even-numbered dimension. For example, the vector [0.3, 0.8, 0.4, 0.2, 0.6, 0.1]^*T*^ will become [1.1, 0.6, 0.7]^*T*^ after processing. This is just a way to preserve the original information as much as possible while compressing the dimensions. Transfer learning is to transfer the trained model parameters to the new model to help the new model training. In other words, take the model developed for task A as the starting point and reuse the process used to develop the model for task B. We refer to the idea of transfer learning to transfer the rating prediction task model to the top-k task. Since the two tasks are strongly correlated, transfer learning allows us to share the learned model parameters to the new model in a way that speeds up and optimizes the learning of the model, rather than learning from scratch as most networks do.

## Experiments

In this section, we provide three metrics to evaluate the proposed BC-PMLP with SVD++ as the underlying model. The experimental results demonstrate evidence of significant improvement over multiple classic and competitive baseline methods. The following text also contains some additional experiments to verify the effectiveness of the proposed method and parameter sensitivity.

### Comparison algorithms

In order to verify the validity of BC-PMLP, we selected eight classic or state-of-the-art methods as comparison algorithms, and SVD++ was adopted as the underlying model.

- SelfCF [47]: Self-supervised Collaborative Filtering framework, which focuses on augmenting the output embeddings generated by backbone networks, and is proposed in 2021. SelfCF can be easily applied to other CF models. Following the experimental design in [47], we adopt Selfed-lightGCN as a comparison, which takes LightGCN as the CF model [42].
- NGCF [41]: Neural Graph Collaborative Filtering, a state-of-art framework proposed in 2019, which exploits the user-item graph structure by propagating embeddings.
- BPR-MF [28]: Bayesian Personalized Ranking based on Matrix Factorization, one of the most famous and effective algorithms in recommender system proposed in 2012.
- NCF [22]: Neural Collaborative Filtering, an excellent framework among the algorithms using implicit feedback for recommendation, which is proposed in 2017.
- DMF [21]: Deep Matrix Factorization for recommender system. It is proposed in 2017, considering both explicit and implicit interactions, and update parameters with a newly designed loss function.
- SVAE [43]: Sequential Variational Autoencoders for collaborative filtering, which uses timestamps to speculate on the user’s future interaction behavior and is published in 2019. The input and output of this algorithm is different from others, so we adjusted the relevant parameters and deleted the validation set used in the original paper to make the size of test set roughly the same as other algorithms.
- RecVAE [45]: RecVAE introduces several novel ideas to improve Mult-VAE. It uses a separate regularization term in the form of the KL divergence between the actual parameter distribution and the distribution in previous training step preventing instability during training.
- JoVA-H [46]: Joint variational autoencoders, an ensemble of two VAEs, in which VAEs jointly learn both user and item representations and collectively reconstruct and predict user preferences. JoVA can capture user-user and item-item correlations simultaneously. A variant of JoVA, referred to as JoVA-Hinge, includes pairwise ranking loss in addition to VAE’s losses to specialize JoVA further for recommendation with implicit feedback.

In addition, we added two groups of experiments, one of which was a transfer model that only used BC (SVD++_BC), and the other used BC-PMLP but the underlying model was NCF instead of SVD++ (NCF_BC-PMLP).

### Parameter settings

We implemented our transfer model using the Pytorch framework which is available in https://pytorch.org. For PMLP, we adopted the tower structure with a size of 32 → 16 → 8 and Adaptive Moment Estimation (Adam) for faster convergence. It is worth mentioning that the size of the first fully connected layer in PMLP depends on the output dimension of the embedding layer. In our experiments, for Netflix, the learning rate, number of iterations and regulation rate of the BC module are 0.001, 160 and 0.0001; for MovieLens-1m and FilmTrust, the learning rate, number of iterations and regulation rate of the BC module are 0.001, 200 and 0.0001, respectively; for Yelp, the learning rate, number of iterations and regulation rate of the BC module are 0.01, 40 and 0.0001. The dimensions of vectors extracted by SVD++ and NCF were both 32 and the number of steps for SVD++ training is 100 for MovieLens-1m and Netflix. For FilmTrust and Yelp, the dimensions of vectors extracted by SVD++ and NCF were both 25, the number of steps for SVD++ training is 20. The code is publicly available at https://github.com/lvan-cn/BC-PMLP.

Through conducted experiments, we believe that 0.9 is the empirical value of *α* for achieving better experimental performance, which means that the importance of explicit ratings is lower than that of implicit interactions.

### Evaluation metrics

Since the main purpose of BC-PMLP is to transform the rating prediction problem into Top-*k* recommendation, we adopt the following three commonly-used Top-*k* evaluation metrics. Note that historically most literature considered error metrics (RMSE, MAE) for evaluation purposes. However, such classical error criteria do not really measure top-N performance [48]. Consequently, several ranking metrics have been proposed in the last two decades and were adopted to evaluate Top-*k* recommendation tasks. The present work shows the evaluation results for the most commonly used ranking metrics.

- *precision*: Percentage of correctly recommended items in the prediction list. If the item that the user likes is on the recommended list, then that item is correctly recommended. It is a metric that measures the proportion of satisfying recommendations made by the recommender system, indicating the quality of recommendations made with an emphasis on the success of the recommendations. Precision at *k* is the proportion of recommended items in the top-*k* set that are relevant.
- *NDCG*: Normalized discounted cumulative gain, which is used to measure the quality of ranking. It will be higher when items with higher relevance appear at a more forward position of the recommendation list.
- *HR*: Hit ratio, the percentage of users that have at least one correctly recommended item in prediction list.

For all the metrics, the larger value indicates the better performance.

### Experimental results

#### The effectiveness of BC-PMLP

**Fig 3:**
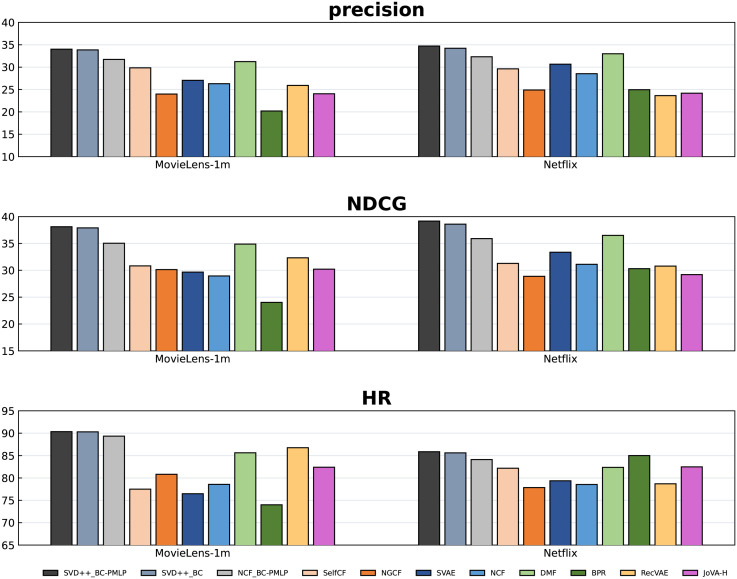
The Top-10 performance of all algorithms on ML-1m and Netflix.

**Fig 4:**
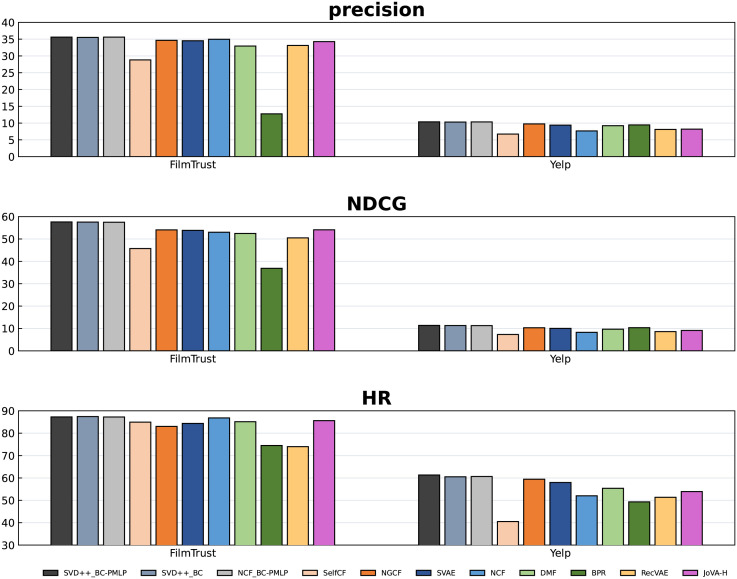
The Top-10 performance of all algorithms on FilmTrust and Yelp.

Figs 3 and 4 show the Top-10 recommendation performance of BC-PMLP based on SVD++ and eight comparison experiments. It can be observed that BC-PMLP has a comprehensive improvement over other algorithms. On the ML-1m, *precision*, *NDCG*, and *HR* have increased by at least 2.78%, 3.24%, and 3.6% respectively (Refers only to the results compared with the eight baselines). For Netflix, we obtain 1.73%, 2.67%, 0.86% improvements of *precision*, *NDCG* and *HR* respectively. For FilmTrust, we obtain 0.64%, 3.53%, 1.65% improvements of *precision*, *NDCG*, and *HR* respectively. For Yelp, we obtain 0.62%, 1.02%, 1.89% improvements of *precision*, *NDCG* and *HR* respectively.

To make a more accurate and comprehensive comparison, we perform each algorithm in cases of Top-5, Top-10 and Top-20. Tables 3–6 provide all the detailed experimental results, where the best performance in each column is marked in bold. From these tables, it can be found that BC-PMLP consistently outperforms all the baselines in most cases. In particularly, the satisfactory *NDCG* performance of SVD++_BC-PMLP and SVD++_BC shows that BC can successfully modify the original distribution, which indicates the success of transfer learning idea. An interesting phenomenon is that the smaller recommendation scale, the stronger superiority of BC-PMLP, which indicates that BC-PMLP can transform and strengthen the information extracted from the underlying model, but its ability of broad learning is limited. According to the main information, BC-PMLP can make more accurate recommendations with a small scale. The increase of the recommendation scale requires the completeness descriptions for all features, rather than the accuracy of some features, so the superiority of BC-PMLP is slightly weakened. BC-PMLP relies on having a sufficient amount of training data to accurately learn user preferences and item characteristics.

**Table 3:** Numerical results recommended by Top-5, Top-10, and Top-20. Note that the numbers are percentage numbers with ‘%’ omitted.

	MovieLens-1m								
	Top-5			Top-10			Top-20		
	*precision*	*NDCG*	*HR*	*precision*	*NDCG*	*HR*	*precision*	*NDCG*	*HR*
SVD++_BC-PMLP	**39.34**	**41.32**	**82.22**	**34.01**	**38.12**	**90.35**	28.14	**36.46**	95.23
SVD++_BC	39.13	41.03	82.05	33.85	37.9	90.3	**28.22**	36.44	**95.3**
NCF_BC-PMLP	36.02	37.45	79.16	31.73	35.04	89.34	26.82	34.11	95.2
SelfCF	31.51	32.09	67.18	29.85	30.82	77.5	26.88	28.8	85.45
NGCF	27.82	30.38	68.32	23.99	30.13	80.82	20.06	33.15	89.77
BPR-MF	30.63	32.12	65.70	27.05	29.67	76.48	22.92	27.86	84.91
NCF	29.85	31.22	67.18	26.3	28.95	78.56	22.33	27.54	86.97
DMF	35.81	37.55	75.57	31.23	34.88	85.62	26.07	33.31	92.21
SVAE	23.2	24.96	62	20.2	24.03	74	20.2	24.66	83.5
RecVAE	30.56	33.88	75.33	25.91	32.33	86.75	20.97	32.93	93.05
JoVA-H	29.15	32	71.91	24.04	30.21	82.4	19.1	30.44	89.73

**Table 4:** Numerical results recommended by Top-5, Top-10, and Top-20. Note that the numbers are percentage numbers with ‘%’ omitted.

	Netflix								
	Top-5			Top-10			Top-20		
	*precision*	*NDCG*	*HR*	*precision*	*NDCG*	*HR*	*precision*	*NDCG*	*HR*
SVD++_BC-PMLP	**39.73**	**42.13**	**77.51**	**34.72**	**39.17**	**85.86**	**28.86**	**37.06**	**91.57**
SVD++_BC	39.21	41.53	77.22	34.22	38.59	85.61	28.55	36.62	91.48
NCF_BC-PMLP	36.14	38.09	74.17	32.32	35.9	84.11	27.54	34.55	90.68
SelfCF	32.57	33.02	72	29.62	31.29	82.17	26.01	29.83	90.35
NGCF	26.7	28.77	65.9	24.89	28.88	77.84	20.4	31.79	86.39
BPR-MF	34.22	35.59	69.62	30.66	33.36	79.36	26.19	31.73	87.14
NCF	31.85	33.14	67.5	28.54	31.11	78.54	24.26	29.54	86.43
DMF	37.47	39.3	73.26	32.99	36.5	82.37	27.77	34.56	88.94
SVAE	30.7	33.19	75	24.95	30.31	85	18.5	28.72	88.5
RecVAE	28.77	31.7	68.19	23.63	30.78	78.69	18.77	30.54	85.55
JoVA-H	28.82	31.4	71.66	24.17	29.2	82.48	19.33	29.54	90.07

**Table 5:** Numerical results recommended by Top-5, Top-10, and Top-20. Note that the numbers are percentage numbers with ‘%’ omitted.

	FilmTrust								
	Top-5			Top-10			Top-20		
	*precision*	*NDCG*	*HR*	*precision*	*NDCG*	*HR*	*precision*	*NDCG*	*HR*
SVD++_BC-PMLP	**42.65**	**52.7**	77.27	**35.62**	**57.62**	87.27	20.37	**62.33**	**92.99**
SVD++_BC	42.56	52.68	**77.35**	35.53	57.55	**87.44**	20.36	62.29	92.99
NCF_BC-PMLP	42.44	52.41	70.09	35.61	57.48	87.26	20.37	62.18	92.91
SelfCF	32.1	39.28	74.01	28.82	45.73	84.94	21.14	54.49	90.85
NGCF	40.35	48.8	73.59	34.66	54.08	84.53	20.09	59.08	91.37
BPR-MF	40.47	48.9	75.38	34.53	53.86	84.62	19.92	58.97	91.54
NCF	39.93	47.11	73.76	34.98	53.03	85.3	20.2	58.26	92.05
DMF	37.93	47.63	75.93	32.96	52.46	85.15	19.68	58.44	90.45
SVAE	15.5	29.14	56	12.75	36.88	74.5	8.98	43.76	89.5
RecVAE	35.6	45.92	65.33	33.13	50.51	74	18.6	54.31	78.67
JoVA-H	40.74	49.17	75.04	34.28	54.09	85.62	**21.46**	59.52	92.42

**Table 6:** Numerical results recommended by Top-5, Top-10, and Top-20. Note that the numbers are percentage numbers with ‘%’ omitted.

	Yelp								
	Top-5			Top-10			Top-20		
	*precision*	*NDCG*	*HR*	*precision*	*NDCG*	*HR*	*precision*	*NDCG*	*HR*
SVD++_BC-PMLP	**12.21**	**12.88**	**44.61**	**10.36**	**11.38**	**61.32**	**9.19**	**11.21**	**79.92**
SVD++_BC	11.83	12.66	44.34	10.3	11.31	60.51	9.1	11.12	79.38
NCF_BC-PMLP	12.13	12.69	44.47	10.35	11.27	60.65	9.1	11.08	78.98
SelfCF	7.37	7.53	26.96	6.73	7.31	40.52	5.88	7.53	54.26
NGCF	10.54	11.05	40.7	9.74	10.31	59.43	7.83	9.66	74.8
BPR-MF	10.32	10.85	38.41	9.39	10.02	57.95	7.96	9.65	73.99
NCF	8.46	9.06	34.1	7.66	8.29	52.02	6.7	8.06	68.6
DMF	11	11.49	37.87	9.22	9.69	55.39	8.69	10.24	72.91
SVAE	11.5	11.64	38.36	9.45	10.36	49.32	7.53	10.31	65.75
RecVAE	8.38	8.49	35.14	8.11	8.61	51.35	7.3	10.62	72.97
JoVA-H	9.46	9.55	36.39	8.19	9.1	53.91	7.64	9.09	72.64

There are some worthy of discussion results on the experiments of MovieLens-1m, that is, using BC independently achieves better results than using BC-PMLP with a tiny gap. After analysis, it is concluded that the combination of BC and PMLP is sensitive to the balance factor *α*, which is empirically set to 0.9 when we test on MovieLens-1m. In order to reduce the complexity of experiments, we continue to adopt this experience when performing algorithms on Netflix, FilmTrust and Yelp. But it is undeniable that BC is largely dominant in transfer recommendation. Quadruple-based training brings BC a strong ability to characterize users and items, so the focus on explicit ratings of PMLP is the icing on the cake. Therefore, in most cases, we prefer to use BC independently instead of BC-PMLP, because the cost of constructing prediction matrices and training perceptron parameters cannot be ignored.

In addition, the performance of NCF_BC-PMLP verifies the certain versatility of BC-PMLP. Taking NCF as the underlying model we can also obtain better performance than NCF itself, this result proves that BC-PMLP can even be extended to Top-*k* prediction models, although the performance of it is still far from SVD++_BC-PMLP.

However, for sparse recommendation data set that there are few interactions available for users or items, the algorithm is not able to learn the underlying patterns very well, leading to not very good recommendations. Sparse data sets, which contain very few interactions or ratings, not provide enough information for BC module to very precisely capture user preferences or identify relevant item features.

#### Impact of initialization

Previous experiments have proved that the transferred information with modified distribution can indeed work in Top-*k* problem, but readers may question that the performances are all attributed to the modification of distribution (BC-PMLP), and have nothing to do with the transfer of information. To this end, we conduct extra experiments with only information transfer, without BC-PMLP, to verify whether pure transfer learning makes sense in recommender systems.

**Fig 5:**
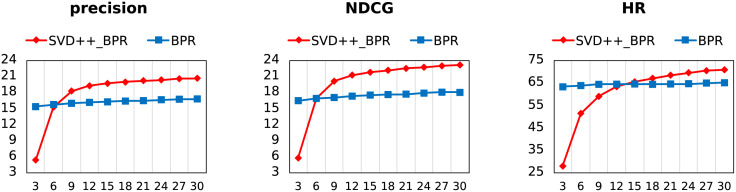
The performance of SVD++_BPR and BPR on MovieLens-1m after every three training epochs.

**Fig 6:**
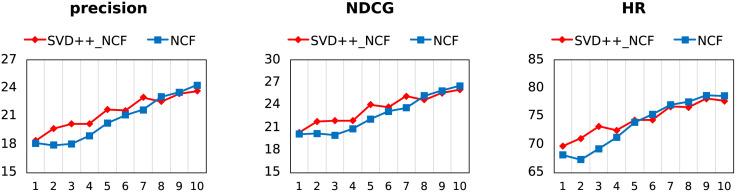
The performance of SVD++_NCF and NCF on MovieLens-1m after every training epoch.

As shown in Figs 5 and 6, BPR-MF initializes with feature vectors extracted in SVD++ (SVD++_BPR) instead of Gaussian random numbers (BPR). The difference between SVD++_NCF and NCF is also the same. All parameters, including learning rate, regularization rate and batch size, are kept consistent. We take observations of the first ten epochs for NCF and every three epochs for BPR.

From the figures, transfer learning has obvious benefit for BPR, and no much for NCF. We give the following explanations: BPR is initialization-sensitive, because there are no other parameters to be trained except feature vectors, and the original vectors carrying effective information make SVD++_NCF slightly better; but a large number of extra parameters included in NCF hide the initialization sensitivity for embedding layers, resulting in almost no difference between transfer or not.

An additional explanation is for the inferior performance of SVD++_BPR to BPR at first three epoch. Gaussian distribution contributes significantly to the performance of BPR-MF at initialization. According to the central-limit theorem, Gaussian random numbers ensure that the initial distribution is consistent with the behavioral characteristics of the entire dataset, that is, the Gaussian distribution describes the characteristics of the sample population, although its description of the individual may be inaccurate. The transfer vectors will not provide the same guarantee, and their descriptions of individuals are serving for rating prediction tasks.

#### Utility of quadruple training

The quadruple training is crucial for the representational ability of BC. But the above content proves either its theoretical superiority or the ability for processing transferred information. Therefore, we also conduct experiments to observe superiority of the quadruple training method alone. Except for the different construction methods of the training pairs (SVD++_BC and SVD++_BPR), the remaining details are exactly the same, and SVD++ is also adopted as the underlying model.

**Fig 7:**
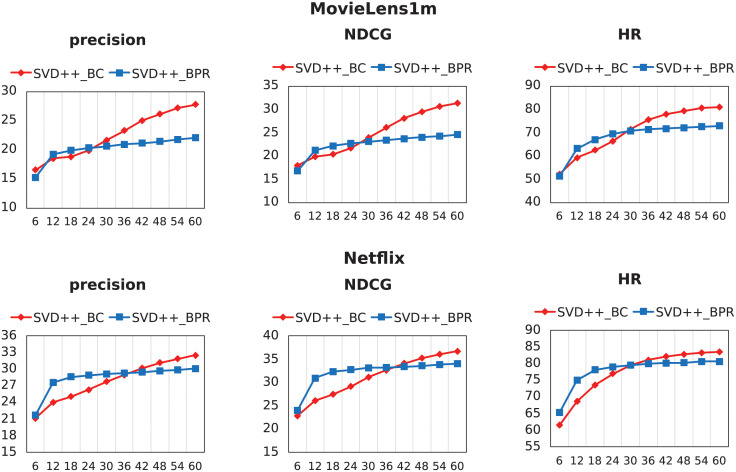
The performance of SVD++_BC and SVD++_BPR after every six training epochs.

As shown in Fig 7, the quadruple training method has achieved comprehensive improvements in terms of convergence speed and final performance. The poor performance at the beginning of training is due to the two negative elements contained in a quadruple. Before positive element information is fully learned, more negative elements will naturally reduce the effectiveness.

#### Sensitivity of *α*

**Fig 8:**
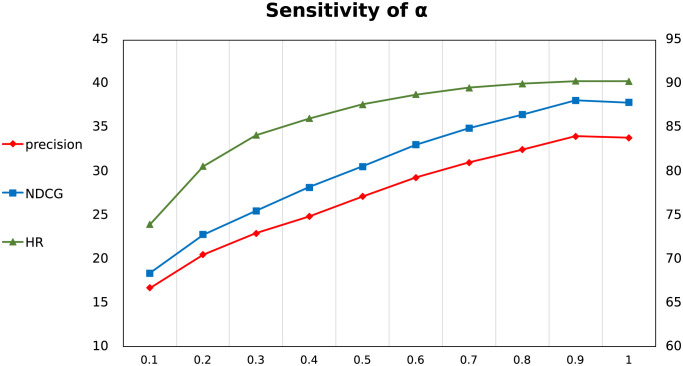
The performance on MovieLens-1m with different *α* values.

As the crucial factor balancing recommendation results of BC and PMLP, *α* largely influences the final performance, which also indicates the difference in importance between implicit and explicit ratings. As shown in Fig 8, we test *α* value from 0.1 to 1 on MovieLens-1m, and finally get a better choice of 0.9 for *α*. The trends of three metrics also support our previous statement: BC is largely dominant in transfer recommendation.

## Conclusion

In this paper, we have introduced how to apply transfer learning ideas to recommender system to associate rating prediction and Top-*k* task. Specifically, we proposed a Bayesian Converter (BC) to learn the implicit interactions, a Prediction based Multi-Layer Perceptron (PMLP) to concentrate on explicit ratings, and adopted a balance factor for weight balance. The transfer ideas, quadruple training, etc. contained in BC-PMLP can be independently applied to other algorithms. Finally, sufficient experiment results showed the effectiveness of BC-PMLP based on SVD++, and we also analyzed the conditions for knowledge transfer, and the utility of quadruple training method used in BC.
